# Medical Controversy

**Published:** 1857-04

**Authors:** 


*Medical Controversy.*

The two medical journals published at Detroit, Michigan (the *Peninsular* and the *Independent*), have been at war with each other until they have begun to publish certificates, or letters of sympathy and approval from their respective friends. This is certainly no very flattering indication on the part of either, and we would with all kindness advise them to heed the dictation of the *American Medical Gazette*, whose editor, Dr. D. M. Reese, has authoritatively commanded them to hush their belligerent passions and in future to keep the peace.

